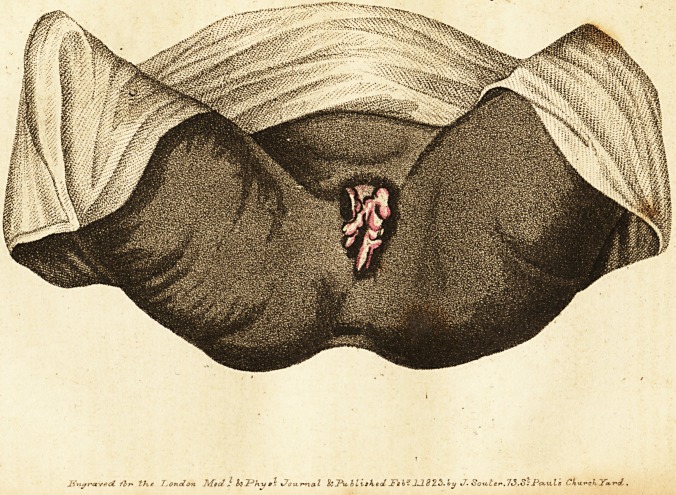# Two Cases of Cæsarean Operation, One of Which Proved Successful

**Published:** 1823-02

**Authors:** John J. U. Van Buren


THE LONDON

### Medical and Physical Journal.

2 OF VOL. XLIX.] FEBRUARY, 1823. [NO. 288.

For many fortunate discoveries in medicine, and for the detection of numerous errors, the world is indebted to the rapid circulation of Monthly Journals; and there never existed any work, to which the Faculty, in Europe and America, were under deeper obligations, than to the Medical and Physical Journal of London, now forming a long, but an invaluable, series.—RUSH.'

### ORIGINAL COMMUNICATIONS,

### SELECT OBSERVATIONS, &c.

ART. I.—*Two Cases of Cæsarean Operation, one of which proved successful.* By JOHN J. U. VAN BUREN. Communicated by Dr. CARSON, of Liverpool.—[With an Engraving.]

ON the 27th of April, 1820, Dr. Doty was called to visit a slave, named Beneba, belonging to William's Hope estate. She was five feet seven inches in height, aged thirty-five years. He was informed by the attendants that she had been nearly sixty hours in labour; that the waters had been discharged, almost at its commencement; and that the pains had, at times, been very severe. He found the pulse regular, the breathing natural, and no symptoms of exhaustion. On examination *per vaginam*, he found the impeded labour occasioned by a peculiar malformation of the pelvis, occasioned evidently by disease of the bones. The small diameter of the pelvis,—viz. between the pubis and os sacrum,—was so narrow as not to admit, with the utmost pressure, the introduction of three fingers: the space was certainly not two inches. From the symphisis pubis a tuberosity projected, about an inch and a half towards the os sacrum. Its base transversely, in a line with the rami, appeared to be an inch and a half, and perpendicularly, or in a line with the symphisis, appeared to be about two inches. The os sacrum was much contracted towards the pubis; the transverse diameter of the pelvis was sufficient to have allowed the passage of the child. The child was alive; the os tincæ well dilated; the head presenting naturally. Dr. Doty was informed that this was the patient's sixth child; that she had been delivered of four dead children, all of whom were born without surgical assistance, except the last, on which occasion, I found, she had been in labour three days; the child was dead, but there was no difficulty in removing it without instruments. It was evident to Dr. Doty that the tuberosity must be an exostosis of the bone, which had been formed since her last delivery; and, the Cæsarean operation being indispensably necessary, he immediately requested my attendance.

After having attentively examined the state of the pelvis, and found the malformation such as to render it impracticable to introduce any instrument for the removal of the child, I did not hesitate in coinciding with Dr. Doty in the necessity of the immediate performance of the Cæsarean section; being of opinion that, where the state of the pelvis is such as to prevent the possibility of the child's passing, no time should be lost, from the firmest persuasion that lives would be saved by the early resort to the operation, instead of waiting (as is too frequently the case) until the patient becomes exhausted by useless efforts to expel the child. The patient, instruments, and dressings, being ready, I operated in the following manner:—Having placed the patient on her back on the table, with a common scalpel I made an incision through the integuments, from the umbilicus to the pubis, so as to expose the linea alba. An opening was then made at the superior part of the incision, through the aponeurosis of the linea alba, into the cavity of the abdomen. Introducing two fingers as a defence against wounding any of the viscera, with a curved bistoury the linea alba and peritoneum were divided, the full extent of the first incision. The uterus immediately presented itself; an inspection, to ascertain the situation of the placenta, being previously made with all possible dispatch. An incision was made into the superior part of the fundus uteri, and extended about seven inches, when the fœtus and placenta were extracted as speedily as possible. In the instant of removing the fœtus, a prolapsus of the intestines took place, but they were immediately returned, and kept *in situ* by Dr. Doty. The lips of the external wound were brought in contact by sutures and adhesive plaster, care being taken not to include the peritoneum in the sutures. The eighteen-tail bandage was used, the patient put to bed, and an anodyne given.

Previous to operating, I weighed well in my mind the importance of the operation, the responsible situation I stood in regarding the life of the patient, and my own reputation as a surgeon. In considering these circumstances, I did not hesitate in adopting the plan pursued; discarding the usual method, as retarding the operation, by the taking-up of arteries, and endangering the life of the patient by the freer admission of air, in consequence of muscular contraction.

Particular care should be taken to prevent a prolapsus of the intestines; and, when it does occur, they should be returned with the greatest dispatch by the assistant: otherwise, it will cause much delay, and expose the contents of the abdomen to the action of atmospheric air,—a circumstance, above all others, to be guarded against.

The patient did not lose more than eight ounces of blood during the operation, which she bore with an astonishing degree of firmness, exhorting her friends not to dishearten her by their lamentations. Every precaution was taken to save the child, which lived for several hours. It was a large-sized child, measuring in length upwards of twenty inches; the circumference of the head was about fourteen inches. The patient slept well during the greater part of the night. Not having passed urine for some time, she was relieved by the catheter. She was free from pain, and had every appearance of doing well, until the morning of the fourth day, when she complained of thirst, had slight fever, but felt no pain or soreness of the abdomen. At noon she complained of griping pain: an enema was ordered, which procured two evacuations, and afforded her relief. The rest of the day she continued free from pain. Her diet consisted of salup, panada, and flour-pap. She slept well this night, without an anodyne.

About two o'clock P.M. of the following day, (the fifth after the operation,) she became restless, and complained of much pain in the lower part of the wound, attended with nausea, inducing efforts to vomit. The pulse was contracted, hard, and upwards of 100; tongue dry. Thirty ounces of blood were taken from the arm, and the following anodyne administered:

℞. Tinct. Opii, m. xl.

Spt. Æth. Nit. ʒss.

Aq. Menth. Sat. ʒx. M.

In about half an hour she found relief, and slept for several hours. Her bowels were kept open by the use of glysters, and the same diet was continued.

On the sixth day, she had no return of pain or nausea, and her appetite was good, until a few hours after the first dressing, (which took place on this day,) when she was attacked with intense pain in the abdomen, attended with vomiting. Her bowels were now perfectly free; the pulse upwards of 100, and, as before, hard and contracted. Sixteen ounces of blood were taken from the arm, and flannels steeped in warm spirits applied to the abdomen. She had a severe rigor. The patient attributed this relapse to the circumstance of the door of her hut (which opened directly opposite her bed,) being incautiously left open for several hours, during which time she was exposed to the draught of a northerly wind. The first bleeding afforded no relief; and no internal remedy could be resorted to, for the stomach rejected every thing. In about three-quarters of an hour, twenty-four ounces of blood were taken from her.

Shortly after this bleeding she found relief, and fell into a sleep of several hours duration. The wound retracted at the inferior part of the incision, for about two inches: it, however, did not assume an unhealthy appearance.

The following day she was much better, and continued doing well until the eighth day, when, unfortunately, the bedstead on which she lay fell down, and gave her a violent and sudden shock. She soon after complained of having received sensible injury from the fall. On the evening of the same day she complained of violent pain in the neck and back, in the course of the spine, with stiffness of the jaws and difficulty of degluti-tion. I visited her about two hours after she was seized. The jaws were much locked, and the spasms increasing rapidly in violence. One drachm of laudanum was administered in a gill of white wine. The neck, back, and throat, were rubbed with warm oil and laudanum. The spasms not abating in half an hour, two drachms of laudanum were administered in burnt brandy. In about a quarter of an hour after the last dose was given, the symptoms abated; but at the expiration of the half-hour the dose was repeated, as before, in burnt brandy, as the patient did not appear to be sufficiently under the influence of the medicine to produce a complete remission of symptoms. Shortly after this she fell into a profound sleep, and the tetanic symptoms did not return. Her diet was now ordered to consist of animal food, and madeira wine, to the extent of three gills, per day. The wound retracted a little more, assumed an un-healthy appearance, and for several days the discharge was sanious and fetid. Stimulant applications were made use of, and in a few days healthy action was renewed. The wound was dressed daily, from the sixth day, until cured.

From this time no material circumstance occurred. On the nineteenth day from the operation she was able to walk about the yard. An elastic roller was ordered to be applied over the abdomen, and worn for some time. The patient continues well to this day, and does field-work with the rest of the negroes on the estate.

*Unsuccessful Case.*

On the 20th of May last (1822), I was sent for to attend a negro woman, (one of the African apprentices,) in labour with her first child; was informed by the attendants, she had been in pain about eight-and-forty hours. Upon examination, found an extensive warty excrescence, uniting firmly the labia nearly their whole extent, admitting the introduction of but one finger into the vagina. Under these circumstances, I advised the re-moval of the woman to town, about the distance of nine miles, with the intention of removing the tumor. As soon as she arrived in town, Doctors Porter and Ross were called in consultation, when it was decided that, considering the indurated nature and situation of the tumor, excessive hemorrhage would attend the excision, and, in all probability, endanger the life of the woman; and that, therefore, the Cæsarean operation was the only alternative. Accordingly I operated immediately, by opening into the cavity of the abdomen, in the course of the linea alba. An incision was made into the uterus, sufficient to have extracted the child; but immediate and powerful contraction of the uterus took place, and prevented for a moment the removal of the child and placenta. An extension of the incision in the uterus was made with all possible dispatch, by which, together with considerable force applied, the patient was delivered of a female child. The wound was immediately closed, and secured by sutures, adhesive plaster, and many-tailed bandage. The patient was put to bed, and an anodyne given. She continued free from pain, was cheerful, and sanguine as to the event, for two days, when her bowels became obstinately costive, and the abdomen very tense. A saline cathartic was administered, without effect. Injections were given every two or three hours, which opened her bowels on the third day. The fourth, she continued to do well. On the fifth, her stools became frequent and watery: ordered port wine and Indian arrow-root, with anodyne injections. The discharge from the bowels was arrested for a few hours; but, owing to the want of attention in the regular administration of wine and nourishment during the night, the discharge from the bowels returned with increased violence, under which she sunk, and died on the sixth day after the operation.

Dressings were removed twice after the operation.

From the time of the operation to her death she was free from fever and any untoward symptom, until the affection of the bowels came on, and terminated her existence. The child is in good health.

I received great assistance from Doctors Porter and Ross, in keeping the contents of the abdomen *in situ*, and in the subsequent treatment of the patient.

This case shows how favourable this climate is in operations of the most formidable nature, and that nothing is to be apprehended from active inflammation: on the contrary, in all operations, stimulant applications should be made use of to the lips of the wound, to produce adhesive inflammation; the patient put immediately on a nourishing diet, and wine given freely. Wherever I have adopted this mode of treatment, it has been attended with the most happy result; and I am persuaded, were the practice universally adopted in warm climates, operations would be attended with more success.

*Tortola; May 29th,* 1822.

## Figures and Tables

**Figure f1:**